# Case of Resuscitation from Apparent Death from Chloroform

**Published:** 1875-03

**Authors:** A. Eubank

**Affiliations:** Alabama.


### ARTICLE IV.

### *Case of Resuscitation from Apparent Death from Chloroform.*

AS RELATED BY DR. A. EUBANK, OF ALABAMA.

A young lady, between fifteen and twenty years of age, came to his office accompanied by her physician, for the purpose of having a tooth removed. Having expressed a desire that the physician should administer chloroform, he did so, and the tooth was extracted without difficulty. About five minutes afterward, and when consciousness had returned, respiration suddenly ceased, and though the chair was thrown back to bring the body in a more horizontal position, and the dress was loosened, yet no sign of returning life was manifest. In this emergency two other physicians were sent for, while Nelaton's method of inverting the body was resorted to. The effect was prompt and satisfactory, respiration at once being resumed, and continued during the time the patient was kept in that position; but immediately upon the resumption of the horizontal condition, respiration at once ceased again. The inversion was repeated with the same satisfactory results, respiration commencing promptly and continuing for some ten minutes, when on the body being again placed in a recumbent posture, breathing again ceased. In the interim, on the advice of the medical men present, Granville's lotion was applied to the spine, and the feet immersed in hot water, but with no apparent good results. For the third time the inversion was resorted to, and the patient kept in it for some ten minutes or more, when respiration seeming to be fully established, the body was placed in a recumbent position once more, and no return of the grave symptoms was seen.

During all this time there was spasmodic closure of the jaws, and preventing access to the tongue had it been in the way.

The case is one of interest, as proving in a conclusive manner the efficacy of this method of resuscitation from apparent death by chloroform, and has suggestive value.

---

